# Polyelectrolytes in Hot Melt Extrusion: A Combined Solvent-Based and Interacting Additive Technique for Solid Dispersions

**DOI:** 10.3390/pharmaceutics11040174

**Published:** 2019-04-10

**Authors:** Felix Ditzinger, Catherine Dejoie, Dubravka Sisak Jung, Martin Kuentz

**Affiliations:** 1 Department of Pharmaceutical Sciences, University of Basel, 4056 Basel, Switzerland; felix.ditzinger@fhnw.ch; 2 Institute of Pharma Technology, University of Applied Sciences and Arts Northwestern Switzerland, Hofackerstr. 30, 4132 Muttenz, Switzerland; 3 European Synchrotron Radiation Facility, 38000 Grenoble, France; catherine.dejoie@esrf.fr; 4 Department of Chemistry, University of Zurich, 8057 Zurich, Switzerland; dubravka.sisak@chem.uzh.ch

**Keywords:** polyelectrolytes, amorphous solid dispersions, hot melt extrusion, polyelectrolyte excipient matrix

## Abstract

Solid dispersions are important supersaturating formulations to orally deliver poorly water-soluble drugs. A most important process technique is hot melt extrusion but process requirements limit the choice of suitable polymers. One way around this limitation is to synthesize new polymers. However, their disadvantage is that they require toxicological qualification and present regulatory hurdles for their market authorization. Therefore, this study follows an alternative approach, where new polymeric matrices are created by combining a known polymer, small molecular additives, and an initial solvent-based process step. The polyelectrolyte, carboxymethylcellulose sodium (NaCMC), was tested in combination with different additives such as amino acids, meglumine, trometamol, and urea. It was possible to obtain a new polyelectrolyte matrix that was viable for manufacturing by hot melt extrusion. The amount of additives had to be carefully tuned to obtain an amorphous polymer matrix. This was achieved by probing the matrix using several analytical techniques, such as Fourier transform infrared spectroscopy, differential scanning calorimetry, hot stage microscopy, and X-ray powder diffraction. Next, the obtained matrices had to be examined to ensure the homogeneous distribution of the components and the possible residual crystallinity. As this analysis requires probing a sample on several points and relies on high quality data, X-ray diffraction and starring techniques at a synchrotron source had to be used. Particularly promising with NaCMC was the addition of lysine as well as meglumine. Further research is needed to harness the novel matrix with drugs in amorphous formulations.

## 1. Introduction

The rising number of poorly water-soluble drugs in the development pipelines as well as on the market encouraged the pharmaceutical industry to develop new formulation techniques. One strategy is the formulation of a drug in an amorphous form as a solid dispersion, which normally leads to drug supersaturation upon oral administration to promote absorption [1,2,3,4,5]. Among the different process techniques for the manufacturing of amorphous solid dispersions, hot melt extrusion (HME) and spray drying are the most common methods [5,6]. These two process techniques mostly use a combination of drug and polymeric compound. However, HME formulations currently available on the market utilize only about six of the pharmaceutically accepted polymers or a combination of these [6]. Contemporary research is primarily focused on finding new combinations of well-established polymers with plasticizers and surfactants [7], or even on designing new monomers for novel synthetic polymers that come with the aforementioned multiple development hurdles to reach the pharmaceutical market [8]. Another approach is the fine tuning of the extrusion process by changing screw configuration, temperature profiles or by employing different downstream processing steps [9,10].

Recently, we introduced the approach to molecularly modify a polymeric matrix by interacting excipients [11]. The difference to a classical mixture approach with excipients is that molecular interactions are specifically targeted by design and cannot be facilitated in an extrusion of the physical mixture. In line with this idea, the current study explores the possibility to use selected additives that can interact ionically or via hydrogen bonding to enable HME of a matrix based on the polyelectrolyte carboxymethylcellulose sodium (NaCMC) for the first time.

NaCMC was recently extruded with polydimethylsiloxane as a polymeric mixture to form material for 3D printing [12] or it is occasionally used in spray drying [13]. The polymer shows good water solubility and extensive swelling behavior, which are both interesting properties for a new modified matrix produced by HME.

The concept of formulating ionic substances to produce a semi-solid or even liquid with a lower melting point is a well-known technique of “ionic liquids” and an important pharmaceutical application in the field of lipid-based formulations [14,15]. Recent publications highlighted the positive implications of salt formation on HME [16,17], but primarily for keeping the drug in amorphous form through the formation of ionic interactions [18,19]. Such an approach is of particular interest, since the direct extrusion of neat unprocessed NaCMC is not applicable, because it decomposes at 252 °C instead of having a melting point [20].

Therefore, this paper studies polymeric films of NaCMC in combination with six interacting small molecular additives that were first transformed into a solid excipient dispersion through solvent evaporation. In a second processing step, HME was performed. The solvent evaporation step (involving a medium with a high dielectric constant) enabled targeted ionic interactions between polyelectrolyte NaCMC and the ionizable additive [18]. The main reason, why a solvent evaporation step was conducted prior to extrusion was that the compounds used would not be feasible for extrusion as otherwise neat powders because of their high melting points.

As the first group of coformers to be studied with NaCMC, the basic amino acids, histidine, lysine, and arginine, were chosen, as they have been proven to interact with acidic groups of mostly drugs in various studies and consequently improved formulation properties such as amorphous stability, miscibility and plasticizing effects [21,22,23,24,25,26,27,28,29]. The second group of substances consisted of water-soluble inactive substances, which were also hypothesized to likely form an interaction with NaCMC after solvent evaporation and extrusion. The chosen coformers were urea, meglumine and trometamol (TRIS).

Powder X-ray diffraction (PXRD) was applied to determine the maximum amount of additive that is still feasible for successful miscibility and an extrusion process to form an amorphous product. Two limiting factors had to be considered during the described processing: on the one hand, the unfavorable extrusion properties of NaCMC, which required a high amount of additive to enable the extrusion and on the other hand, the crystalline structure of the additives, which would lead to a crystalline product in high concentrations because of insufficient miscibility. While the preliminary measurements could be carried out using the laboratory diffractometer, conclusive results could only be obtained by using the data collected at a synchrotron source. Namely, to ensure the amorphous formulation, it was necessary to collect high quality PXRD data that is sensitive to extremely low amounts of crystalline phases in the sample. Secondly, to examine the distribution of the additive in the sample, the sample had to be probed on several points, which again required a specific sample stage at a synchrotron source.

Further assessment included thermal analysis by differential scanning calorimetry, which was complemented by hot stage microscopy and hot stage attenuated total reflectance Fourier transform infrared spectroscopy (ATR-FTIR) to show crystallinity and form changes upon heating [18]. The HSM images were used as a complimentary analysis of the thermal miscibility and melting behavior of the evaporates during the extrusion [10,30].

This paper highlights the capability of different small molecular additives to enable the formulation of a polymeric compound, which would otherwise not be suitable for extrusion. Such a combination resulted in the development of a new modified excipient matrix for HME that formulators will find helpful to cope with challenging pharmaceutical compounds.

## 2. Materials and Methods 

### 2.1. Materials

Carboxymethylcellulose sodium salt (low viscosity), urea, meglumine, TRIS, L-lysine, L-aspartic acid, and L-histidine were bought from Sigma Aldrich (St. Louis, MO, USA). Purified water, which was used for the solvent evaporation, was taken from a MilliQ Millipore filter system (Millipore Co., Bedford, MA, USA).

### 2.2. Methods

#### 2.2.1. Preparation of Hot Melt Extrudates

Binary mixtures of NaCMC and the additive (according to the composition given in Section 3.1.1 and Section 3.2.1) were mixed in a mortar and dissolved in MilliQ water in a round bottom flask. Afterwards, the water was removed by a rotary evaporator (Rotavapor Büchi, Flawil, Switzerland), which resulted in a transparent film. This film was cut into smaller pieces and extruded on co-rotating screws with a 9-mm diameter and 180 mm in length in a ZE9 ECO twin screw extruder by ThreeTec (Birren, Switzerland). A screw speed of 80 rpm was applied at a temperature of 130 °C through all three heating zones. The final extrudates were cooled to room temperature and stored in falcon tubes.

#### 2.2.2. Laboratory Powder X-ray Diffraction (PXRD)

Mixtures were studied for their potential amorphous form by PXRD on a D2 Phaser diffractometer (Bruker AXS GmbH, Karlsruhe, Germany) with a 1-D Lynxeye detector. The instrument was equipped with a Ge-monochromator (Cu Kα radiation) providing X-ray radiation at a wavelength of 1.541 Å. During the measurements, a voltage of 30 kV and a current of 10 mA were used. The increment and time per step were set to 0.020 ° and 1 s, respectively. The measurements were scanning a range of 5° to 40° (2θ).

#### 2.2.3. Differential Scanning Calorimetry (DSC)

Samples were further assessed by a differential scanning calorimeter on a DSC 3 (Mettler Toledo, Greifensee, Switzerland). The samples were cut in small pieces and 5 to 9 mg was placed in a 40 μL aluminum pan with a pierced lid. A heating rate of 10 °C/min from −10 °C to 140 °C was applied, while the surrounding sample cell was purged with nitrogen 200 mL/min. Moreover, the combination of heating, cooling and heating cycles was used to fully evaluate the samples. For the assessment of the initial form, the first heating was used. The thermograms and glass transition temperatures (*T_g_*s) were analyzed with the STARe Evaluation-Software Version 16 (Mettler Toledo, Greifensee, Switzerland). All thermograms show exothermic events as upward peaks.

#### 2.2.4. Hot Stage Attenuated Total Reflectance Fourier Transform Infrared Spectroscopy (ATR-FTIR)

A Cary 680 Series FTIR spectrometer (Agilent Technologies, Santa Clara, CA, USA) was used, which was equipped with a heatable attenuated total reflectance accessory (Specac Limited, Orprington, UK) and the control panel 6100+ by WEST (West Control Solutions, Gurnee, IL, USA). The scanning range of 4000–600 cm^−1^ was selected with 1500 scans over a period of 30 min and a resolution of 4 cm^−1^. The heating rate was set to 5 °C/min going from 30 °C to 130 °C. For the evaluation, a spectrum was extracted and evaluated by the software ACD/Spectrus Processor 2016.1.1 (Advanced Chemistry Development, Canada) every minute (i.e., every 5 °C). Every spectrum shows a 5 °C temperature increase going from the front to the back of the figures. The increase of peaks towards higher temperatures in the area of 2000 cm^−1^ is related to the heat implications on the ATR crystal. For the hot stage FTIR analysis, the solvent evaporated films were used, whereas the FTIR spectra at room temperature were recorded from the physical mixture, solvent evaporates, and extrudates.

#### 2.2.5. Synchrotron Powder X-ray Diffraction

X-ray powder diffraction data were recorded at the ID22 beamline at the European Synchrotron Radiation Facility (ESRF, France) using a two-dimensional detector (PerkinElmer XRD 1611CP3) and an incident X-ray energy of 60 keV (λ = 0.20678 Å, Qmax = 24 Å^−1^). A beam size of about 0.5 mm × 0.5 mm was used. Reference samples were packed in 0.7-mm diameter borosilicate capillaries. Extrudate samples were mounted directly on capillary supports and measured as is. In order to minimize any possible radiation damage, samples were cooled down to 100 K using an Oxford Cryosystem Cryostream. To improve the overall statistics, 200 two-dimensional images were recorded (2 s per frame) and averaged. The one-dimensional diffraction patterns were retrieved after integration using the PyFAI software [31]. Five diffraction patterns on five different locations were recorded on each extrudate sample in order to check for heterogeneity.

#### 2.2.6. Hot Stage Microscopy (HSM)

The HSM analysis employed a Leica DMRM at magnifications of 100×, which is also displayed as a scale bar in the images. The microscope was equipped with a temperature-controlled microscope stage from Linkram. This analysis was used for the evaluation of the behavior of the formulation upon heating in the extruder and to complement the DSC analysis [9,17,32]. For a close relation to the extrusion process, the temperature ramp was set from room temperature (RT) to 130 °C. During this ramp, the temperature was kept steady and images were taken at RT, 90 °C, and 130 °C. The obtained images were converted into black and white to highlight the melting process.

## 3. Results and Discussion

### 3.1. Amino Acids as Additives

#### 3.1.1. Characterization of the Formulations

Formulations containing the additives arginine and lysine were found to be amorphous after evaporation as well as extrusion. In contrary, it was not possible to convert histidine to an amorphous form neither with evaporation nor with extrusion. Table 1 highlights the different aspects, which were essential during processing of the formulation such as a qualitative evaluation of technical feasibility during HME. The extrusion was evaluated compared to a standard extrusion of the polymer, PVPVA 64, which is considered arbitrarily as ideal for extrusion. Such extrusion behavior is influenced by melt viscosity, thermoplasticity, and degradation [10].

**Figure 1:**
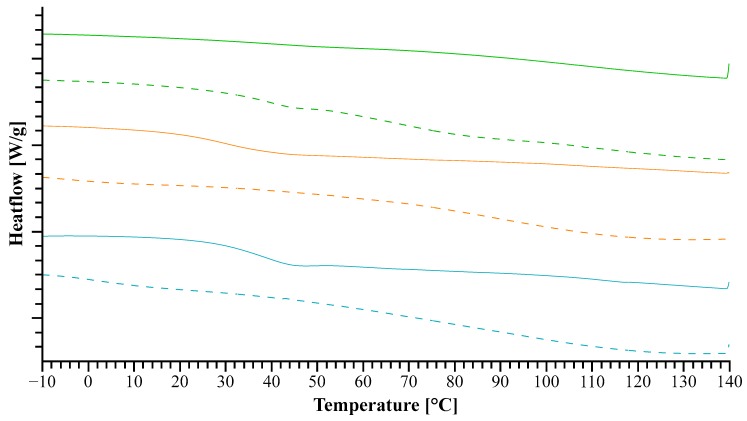
The solid lines represent the differential scanning calorimetry (DSC) thermograms of the extrudates and the dotted lines represent the evaporates which were used for the later extrusion. The amino acids added to sodium carboxymethylcellulose (NaCMC) are arginine (cyan), lysine (orange), and histidine (green).

The optimal amounts of additives necessary to produce an amorphous polymer matrix are presented in Table 1, expressed as loadings in weight/weight as well as the calculated molar fractions of the formulation components. Lysine resulted in the highest amount of additive, which was formulated in an amorphous form in combination with NaCMC, whereas histidine being less feasible for the evaporation and the later extrusion could only be incorporated in the lowest molar ratio used in this study. This is also reflected by very poor extrusion behavior as well as the disappearance of the *T_g_* in the DSC measurements of the corresponding extrudates, which may be explained by recrystallization from amorphous state as crystallinity was found in the extruded histidine formulation (Figure 1). For the above-mentioned table, it has to be mentioned that lower amounts of additive during a previous formulation development were leading to worse extrusion performances, which underlines the insufficient extrusion performance of neat NaCMC.

In detail, the dotted lines in Figure 1, representing the solvent evaporates, show only slight indications of a *T_g_* in all samples. Whereas only the thermograms of extrudates containing lysine and arginine show the presence of a clear *T_g_* in the extrudates (see Table 1). This can be associated with an amorphous form of the additive in the formulation [33] and gives a first indication of formed molecular interactions [22,26]. These two additives also formed more prominent *T_g_*s during the extrusion, which entails a higher amount of amorphous additive in the formulation. Consequently, such a processing was beneficial for the formation of an amorphous modified matrix of NaCMC. However, this still needed further measurements for confirmation.

As mentioned before, the *T_g_* in the histidine extrudates disappeared after extrusion, which suggested that the amorphous form changed during extrusion, leading to a crystalline fraction as indicated by the diffraction peaks in the corresponding PXRD analysis (Figure S3). Although a *T_g_* was detectable for lysine and arginine after the extrusion in the DSC, it has to be kept in mind that the substances used show rather high individual melting points, which would have led to degradation during the thermal measurement. 

Therefore, to obtain high quality data that is sensitive to extremely low amounts of crystalline phase in the sample, it was necessary to perform the diffraction and scattering experiments at a synchrotron source.

**Figure 2:**
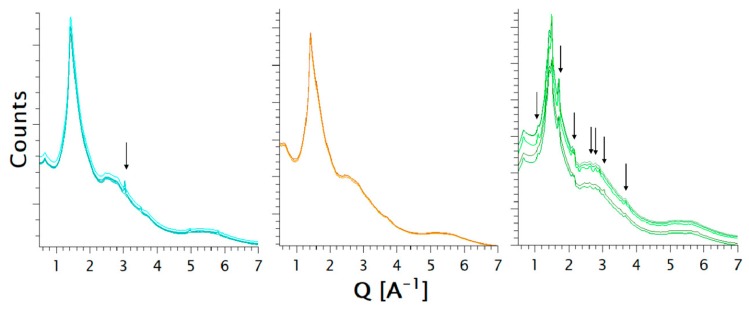
X-ray synchrotron results (i.e., arbitrary counts versus Q vector, Q = 4πsin(θ)/λ) are displayed from the extrudates with amino acid coformers. The amino acids added to NaCMC are from left to right: arginine (cyan), lysine (orange), and histidine (green). Each diffraction pattern corresponds to a measured area in the extrudate.

Thus, synchrotron X-ray diffraction offered a more thorough assessment of the amorphous form to complement the DSC and benchtop PXRD data, which indicated that the raw substances were crystalline except for the polyelectrolyte NaCMC (Figure S1). PXRD data collected at the synchrotron source featured Bragg peaks that could be related directly to the crystallinity of the respective additive. Pronounced crystallinity evidenced in the histidine evaporate was in accordance with the initial X-ray and DSC assessment and was still detectable after extrusion, which is pointed out by the peaks at 1.07 A^−1^, 1.71 A^−1^, 2.11 A^−1^, 2.60 A^−1^, 2.81 A^−1^, 3.06 A^−1^, 3.61 A^−1^ (Figure 2). Moreover, the measurement at five different locations throughout the extrudate showed the inhomogeneous distribution of the crystalline additive in the extrudate (Figure 2), which can potentially lead to more recrystallization. The diffraction pattern of the arginine extrudate indicated a more homogeneous distribution of the additive compared to histidine, although peaks at 3.05 A^−1^ still underline some partial crystallinity of the extrudate, which was detectable neither in the initial benchtop PXRD assessment nor by DSC.

**Figure 3:**
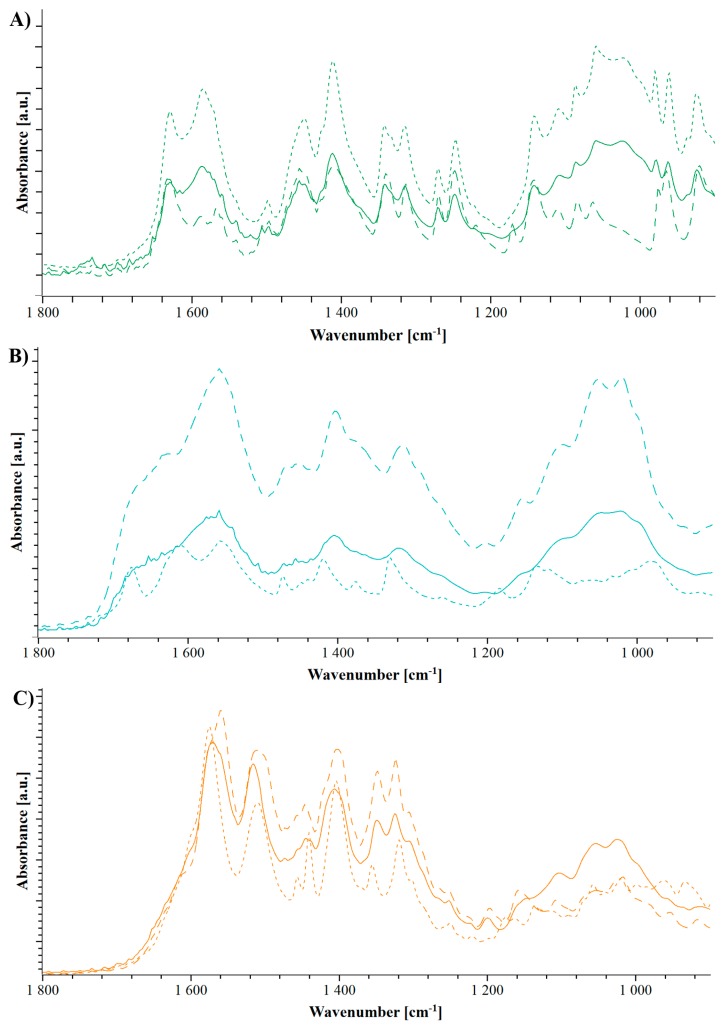
Fourier transform infrared spectroscopy (FTIR) spectrograms of NaCMC and histidine (**A**, green), arginine (**B**, cyan) and lysine (**C**, orange). Dotted lines represent the physical mixture, dashed lines represent the solvent evaporates and the extrudates are shown in solid lines.

The FTIR spectra of arginine/NaCMC in Figure 3B exhibit reduced guanidyl vibrations of arginine at 1675 cm^−1^ and 1614 cm^−1^, which can be associated with the interaction between the ionized arginine side chain and the negatively charged NaCMC [22].

For the coformer lysine, only smaller shifts in the FTIR spectrum are present in the evaporate and the extrudate including the shoulder of the COO^−^ bond at 1607 cm^−1^, which is less pronounced in the extrudate than in the physical mixture [12,34]. In addition, a slight shift and a pronounced broadening of the peaks at 1570 cm^−1^ and 1540 cm^−1^ [35] both highlight the interaction of the carboxylic group of NaCMC (Figure 3C). The analysis of histidine/NaCMC in Figure 3A shows pronounced similarities of the physical mixture and extrudate. This supported the previous findings of the extrusion leading to a change in the solvent evaporate with recrystallization of histidine [21].

As mentioned in the previous section, the NaCMC was completely amorphous prior to processing. Therefore, the observed peak broadening and shifts are related to the amorphization of the additive.

#### 3.1.2. Heat Assisted Characterization

The hot stage microscopy was applied to better understand the processes occurring during the extrusion of the evaporated films and to complement the results of extrusion performance using the different additives. It should be noted that bright structures in the HSM images are not necessarily related to crystallinity as they can also highlight an increase in capillarity of the samples.

**Figure 4:**
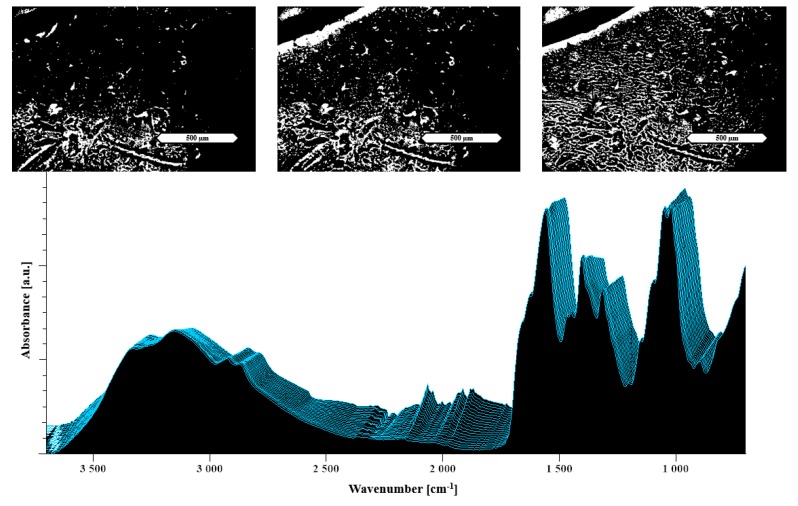
Hot stage microscopy (HSM) images at the top and temperature-resolved FTIR of 33% arginine in NaCMC at the bottom. The images show HSM images taken at RT, 90 °C and 130 °C (from left to right). The displayed scale bar refers to 500 μm. Each spectrum was measured at temperatures from 30 °C (measured first in the front) to 130 °C (in the back) by increasing steps of 5 °C.

Thus, Figure 4 top shows an increasing number of capillaries building up in the polyelectrolyte film containing arginine, which can be directly associated with the positive extrusion performance. In this case, even though the HSM suggests a successful extrusion, as highlighted in the previous section, the arginine extrudate still contained crystallinity. This could be explained by the insufficient mixing behavior of the two excipients, which is underlined by the minimal changes visible in the heat-resolved FTIR. In Figure 4 bottom, only minor changes in the FTIR are visible during the heating.

The evaporated film containing lysine showed no crystals in the microscopic images and small indications of melting in the images taken at 130 °C in comparison to RT (Figure S4). Even in case of minor melting events, the torque in the extruder facilitates the plasticizing and melting of the evaporate during the extrusion. Therefore, the analysis of films represents a kind of “worst case scenario” regarding shear forces. It is still possible to successfully obtain an extrudable amorphous formulation as in the given case of lysine. The heat-resolved FTIR spectra in Figure S4 at the bottom show an increase in the peak at 1560 cm^−1^ and 1516 cm^−1^, which are related to the carboxylic groups of NaCMC [35]. Such an observation can be interpreted as an increase of the interaction between NaCMC and lysine.

**Figure 5:**
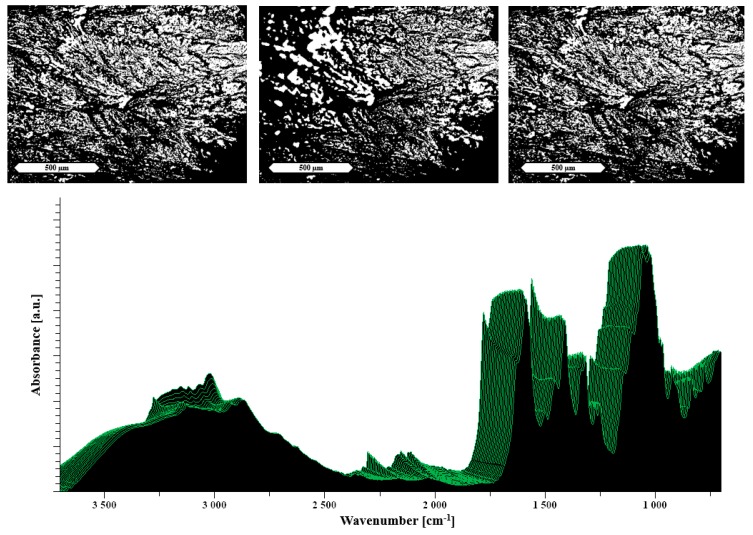
The images at the top show HSM images of histidine/NaCMC evaporated films taken at RT, 90 °C and 130 °C (from left to right). At the bottom, a temperature-resolved FTIR spectrum is shown. The displayed scale bar refers to 500 μm. Each spectrum was measured at temperatures from 30 °C (measured first in the front) to 130 °C (in the back) by increasing steps of 5 °C.

The HSM images of the histidine evaporate at RT showed pronounced crystallinity, which was in accordance with the PXRD diffraction patterns (Figure S2). Moreover, the images taken at the operating temperature of the extruder (130 °C) did not show any reduction in crystallinity or a phase transition, which could be associated with a glass transition. This is supported by the measurable crystallinity and immiscibility in the extrudate (Figure 5 in green) [18].

The thermal evaluation of the evaporated films aligned the prior solid-state characterization as well as the actual behavior in the extruder, meaning the formulations containing arginine and lysine, which were successfully incorporated in a concentration of 33% and 50%, respectively, performed well in the extruder and could only be differentiated by a synchrotron X-ray measurement showing slight crystallinity in the arginine formulation. By contrast, the histidine formulations demonstrated poor melting behavior as well as pronounced crystallinity after extrusion. Moreover, the distribution of histidine was insufficient throughout the extrudate, which leads to differences in the diffraction pattern evidenced by the synchrotron X-ray measurement.

### 3.2. Additives Other than Amino Acids

#### 3.2.1. Characterization of the Formulations

Analogous to previous results, it was necessary to combine solvent evaporation and HME in the mixtures of NaCMC and the further tested coformers. This was suggested by the X-ray diffraction patterns of the formulations following solvent evaporation. The X-ray diffraction pattern of the TRIS/NaCMC solvent evaporates showed Bragg peaks that indicated the presence of TRIS in a crystalline form (Figure S2). However, TRIS was completely transferred into an amorphous form following a subsequent HME step (Figure S3). The urea and meglumine formulations were amorphous after the solvent evaporation and did not recrystallize to a detectable extent based on the benchtop PXRD results (Figure S3).

Table 2 presents a comparison of the maximal amount of additives for which the polymer matrix was kept in an amorphous state. The differences in molar weight have to be taken into account for such an evaluation, leading to a comparable molar fraction of meglumine and urea and a lower loading as well as molar fraction of TRIS (Table 2). This observation was a first indicator of the different technical feasibility of the various additives to obtain suitable modified matrices of NaCMC. A higher loading of TRIS led to crystallinity after evaporation as well as extrusion. Therefore, a lower loading had to be chosen, which resulted in non-ideal extrusion performance as described in the introduction. The additives meglumine and urea could be incorporated at much higher molar ratios and positively influenced the extrusion process.

**Figure 6:**
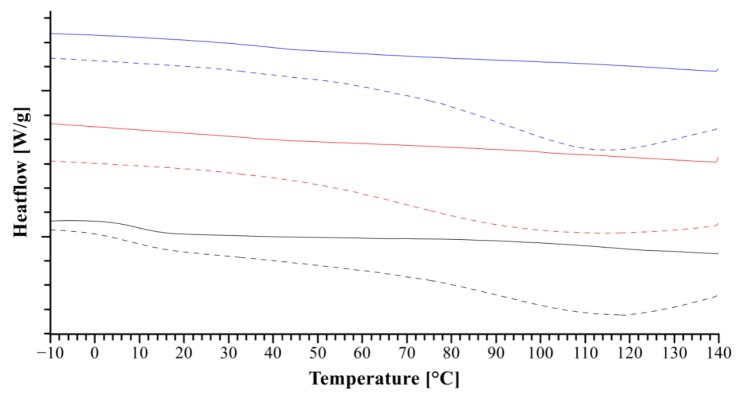
The solid lines represent the thermograms of the extrudate and the dotted lines represent the evaporates, which were used for the later extrusion. The additives used in addition to NaCMC are meglumine (black), TRIS (red) and urea (blue).

The thermograms in Figure 6 indicate the presence of remaining water after the solvent evaporation, given as a broad peak around 100 °C. The *T_g_* of urea can be hardly detected because of small difference in heat capacity at the glass transition and for the TRIS formulation, no *T_g_* could be detected. On the other hand, the extrudate of meglumine shows a rather pronounced *T_g_* and also a shift towards a lower temperature in comparison to all other extrudates, which can be associated with the good miscibility of meglumine and NaCMC [36,37].

**Figure 7:**
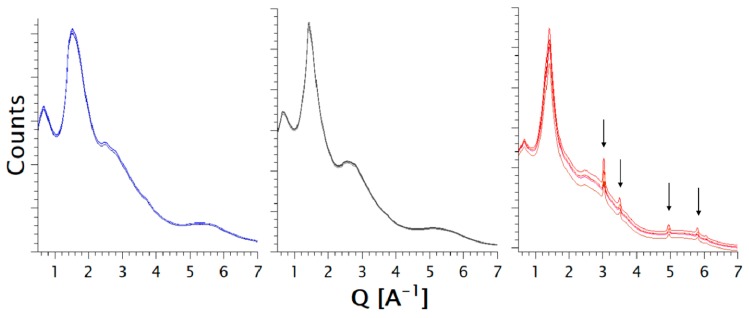
X-ray synchrotron results (i.e., arbitrary counts versus Q vector) of coformers other than amino acids. The additives in addition to NaCMC are: urea (blue), meglumine (black), and TRIS (red). Each diffraction pattern corresponds to a measured area in the extrudate.

All samples were measured at five different areas. However, differences in the patterns can be seen only for the case where TRIS was used as the additive, particularly for differences in scattered intensity and the emergence of Bragg peaks at Q values of 3.03 A^−1^, 3.50 A^−1^, 4.95 A^−1^ and 5.81 A^−1^. By contrast, the patterns of the extrudates containing meglumine and urea present no observable differences in their X-ray synchrotron results (Figure 7). The absence of Bragg peaks in the patterns collected on samples containing meglumine and urea prove that the obtained polymer matrices were fully amorphous at the molar fractions of 0.57 and 0.52, respectively. The PXRD patterns, collected on the sample containing TRIS at the synchrotron source, showed indications of crystallinity, which were not detectable in the patterns of the laboratory diffractometer. Such crystallinity could be a sign of recrystallization after the extrusion as well as residual crystallinity. Both sources of crystallinity are related to the instability of an amorphous form [38].

In the FTIR spectra, the combination of NaCMC and meglumine shows the previously discussed broadening due to the amorphization [39,40]. It can be seen how following solvent evaporation, distinct peaks are observed, which are broadened in one peak at 1000 cm^−1^ and 1400 cm^−1^. Moreover, the previous discussed carboxylic peak at 1570 cm^−1^ of NaCMC is more pronounced and broader, which indicates a change in the intermolecular binding of the polyelectrolyte [34,35].

The FTIR spectrum of urea in line with the spectrum of NaCMC/meglumine shows an increase of the peak at 1580 cm^−1^, which indicates the same interaction with the carboxylic group as meglumine [35]. 

**Figure 8:**
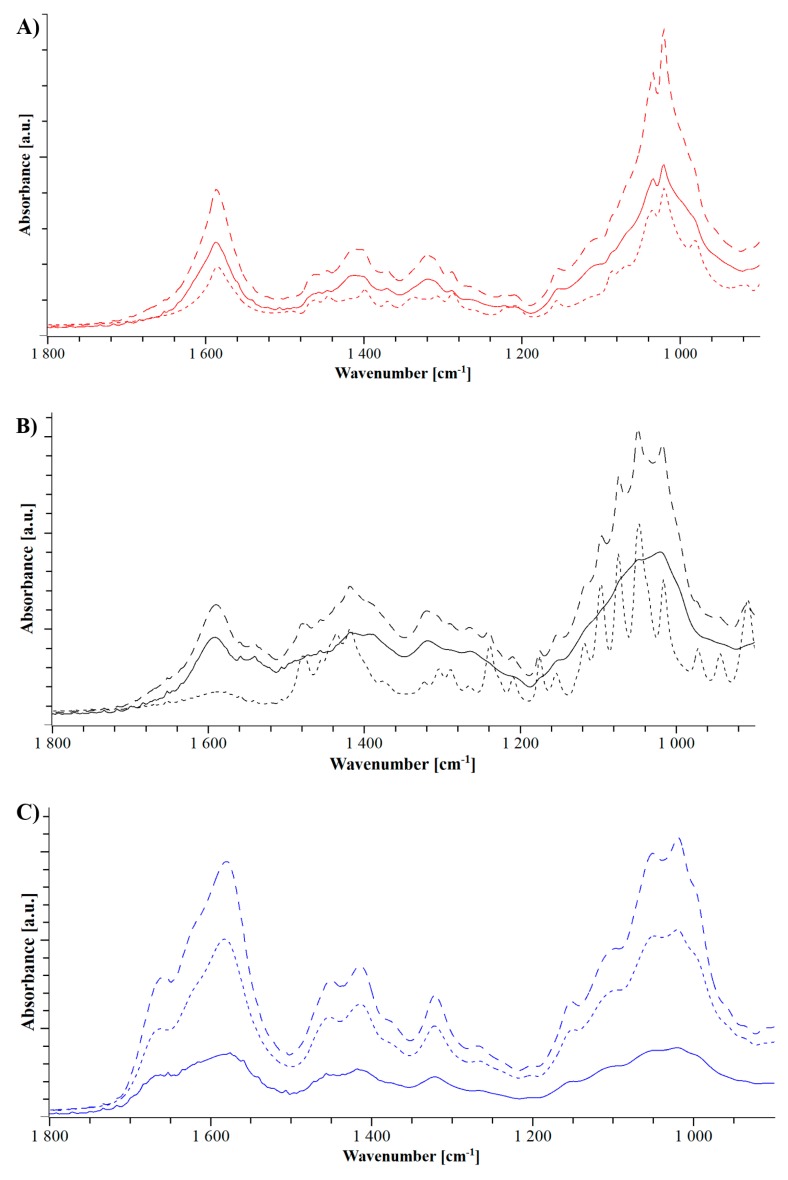
FTIR spectrograms of NaCMC and TRIS (**A**, red), meglumine (**B**, black), and urea (**C**, blue). Dotted lines represent the physical mixture, dashed lines represent the solvent evaporates and the extrudates are shown in solid lines.

The spectrum of TRIS showed specific peak broadening as a result of the amorphous formulation (Figure 8). However, this broadening is overlapping a lot with the peaks formed because of a potential interaction. The increase of broader peak between 1400 cm^−1^ and 1600 cm^−1^ can be interpreted as an indication of an interaction [12,16,41]. However, a further, more sensitive analysis is required for a precise statement about the interaction.

Interestingly, the observed changes in the FTIR spectra indicate a likely change in the hydrogen bonding structure because of the processing rather than the formation of a distinct salt.

#### 3.2.2. Heat Assisted Characterization

**Figure 9:**
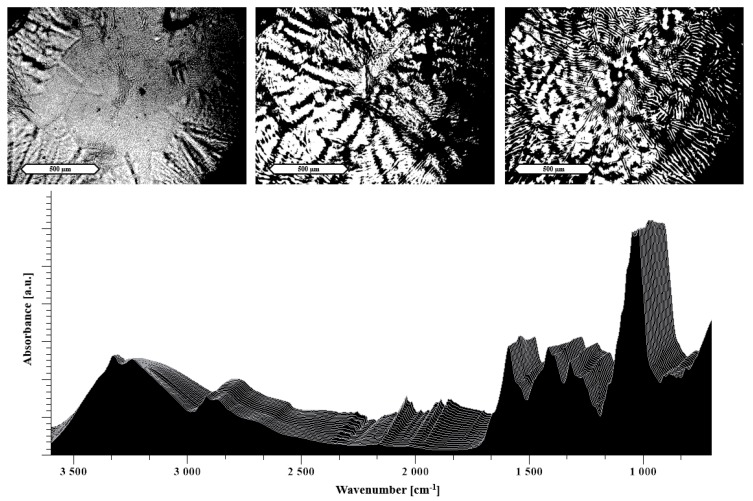
The images at the top show HSM images of 50% meglumine/NaCMC solvent evaporates taken at RT, 90 °C and 130 °C (from left to right). At the bottom, a temperature-resolved FTIR spectrum is shown. The displayed scale bar refers to 500 μm. Each spectrum was measured at temperatures from 30 °C (measured first in the front) to 130 °C (in the back) by increasing steps of 5 °C.

The microscopic images at the top of Figure 9 present the melting process of the meglumine formulation, which can be connected to the thermoplastic behavior in the extruder. The prominent peak broadening around 90 °C in the area above 3000 cm^−1^ is an indicator of a successful amorphization because of differences in the molecular arrangement as well as the near range order [22,40]. This finding is in line with the start of a melting process in the HSM image at 90 °C. Moreover, these findings are in accordance with the performance observed during extrusion of the meglumine formulation.

**Figure 10:**
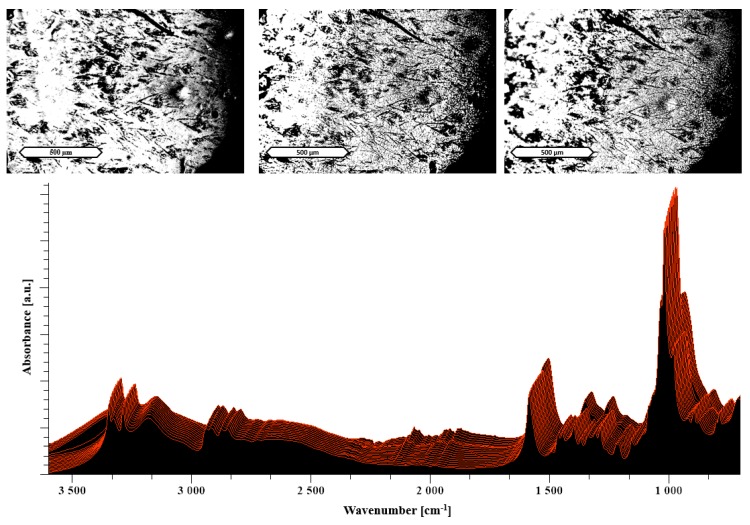
The images at the top show HSM images of 25% TRIS/NaCMC evaporated films taken at RT, 90 °C and 130 °C (from left to right). At the bottom, a temperature-resolved FTIR spectrum is shown. The displayed scale bar refers to 500 μm. Each spectrum was measured at temperatures from 30 °C (measured first in the front) to 130 °C (in the back) by increasing steps of 5 °C.

HSM images of the TRIS formulation show only minor changes. Although, an indication of minor “bubble-shaped” features was recorded that may be associated with small melting events taken place in the formulation (Figure 10). The FTIR spectrum supports the observation of a change with higher temperatures. The broadening of the peaks above 3000 cm^−1^ can not only be associated with the successful amorphization in the TRIS sample [40], as discussed before (Table 1), it furthermore shows the decrease of hydrogen bonding throughout the heating process [35,42], leading to a lack of intramolecular interaction in the NaCMC, thereby resulting in more available interaction sites for TRIS.

As described before, the urea formulation formed an amorphous stable formulation after evaporation and extrusion (Figure S5). The HSM images show a melting process over the temperature range recorded. However, in the heat-resolved FTIR, only minor changes in peak intensity can be observed. In line with Figure 9, this suggests that urea has a plasticizing effect without showing a pronounced interaction with NaCMC. A possible reason for that is the lack of ionizable groups in the urea molecule.

## 4. Conclusions

The application of additives and targeted molecular interactions together with an initial solvent step enabled the extrusion of the polyelectrolyte, NaCMC. Differences in melting behavior and loading highlighted the suitability of the investigated additives to form as a fully amorphous polyelectrolyte matrix after extrusion. The additives, lysine and meglumine, in a concentration of 50% (*w*/*w*), have proven to be beneficial for extrusion and formation of a fully amorphous polymer matrix. Moreover, the application of synchrotron X-ray diffraction helped to further differentiate between the formulations by examining the distribution of the additive throughout the matrix and residual crystallinity in the sample. PXRD data collected at the synchrotron source proved the amorphous state of the lysine, meglumine, and urea formulations compared to arginine and TRIS, for which crystallinity was not detectable by means of benchtop PXRD or DSC.

While recent work has shown that the formulation of an amorphous ionic interaction is possible by hot melt extrusion [17], the current concept presented a not extrudable polymer, which was altered by interacting additives in a modified matrix feasible for HME. We refrained from naming the obtained systems as ionic liquids because this would suggest exclusively ionic coformer interactions with the polymer. Moreover, ionic liquids have an arbitrary defined melting characteristic of <100 °C, which is not required for pharmaceutical application as solid dispersions. However, it is expected that the modified matrices share much of the molecular attractiveness of ionic liquids. Further studies may harness the potential benefits of the solvent evaporates for pharmaceutical HME, reaching from new systems for amorphous drug stabilization over the generation of drug supersaturation to the precipitation inhibition of poorly water-soluble compounds.

## Figures and Tables

**Table 1:** Properties of amino acid / polyelectrolyte matrices.

Additive	Maximum Amorphous Amount	Molar Fraction (Monomeric) *	*T_g_* After Evaporation	*T_g_* After Extrusion	Extrudability **
Amino Acid + NaCMC					
**Lysine**	50% (*w*/*w*)	0.64	30.27 °C	30.62 °C	++
**Arginine**	33% (*w*/*w*)	0.43	35.36 °C	33.15 °C	+
**Histidine**	20% (*w*/*w*)	0.30	36.59 °C	-	- -

* For the calculation, the molar weight of the NaCMC monomer was used. ** Technical feasibility was qualitatively assessed and details are given in the text.

**Table 2:** Properties of the other additive polyelectrolyte matrices.

Additive	Maximum Amorphous Amount	Molar Fraction (Monomeric) *	*T_g_* After Evaporation	*T_g_* After Extrusion	Extrudability **
Other Additive + NaCMC					
**Meglumine**	50% (*w*/*w*)	0.57	5.58 °C	9.18 °C	+
**Urea**	20% (*w*/*w*)	0.52	37.99 °C	40.36 °C	0
**TRIS**	25% (*w*/*w*)	0.42	-	39.18 °C	-

* For the calculation, the molar weight of the NaCMC monomer was used. ** Technical feasibility was qualitatively assessed and details are given in the text.

## Supplementary Materials

The following are available online at https://www.mdpi.com/1999-4923/11/4/174/s1, Figure S1: X-ray diffraction pattern of physical mixtures, Figure S2: X-ray diffraction pattern of the solvent evaporates, Figure S3: X-ray diffraction pattern of the extrudates, Figure S4: Heat-resolved FTIR and HSM images of lysine/NaCMC, Figure S5: Heat-resolved FTIR and HSM images of urea/NaCMC.
