# Supplementary material for: Polyelectrolytes in Hot Melt Extrusion: A Combined Solvent-Based and Interacting Additive Technique for Solid Dispersions
Source: Pharmaceutics. 2019 Apr 10;11(4):174. doi: 10.3390/pharmaceutics11040174 (PMC6523407; doi:10.3390/pharmaceutics11040174)
Supplement: Supplementary file 1 [file pharmaceutics-11-00174-s001.pdf]

pharmaceutics

MDPI

Supplementary Materials

Polyelectrolytes in Hot Melt Extrusion: A Combined Solvent-Based and Interacting Additive Technique for Solid Dispersions

Felix Ditzinger [1,2], Catherine Dejoie [3], Dubravka Sisak Jung [4] and Martin Kuentz [2,*]

[1] Department of Pharmaceutical Sciences, University of Basel, 4056 Basel, Switzerland; felix.ditzinger@fhnw.ch
[2] Institute of Pharma Technology, University of Applied Sciences and Arts Northwestern Switzerland, Hofackerstr. 30, 4132 Muttenz, Switzerland
[3] European Synchrotron Radiation Facility, 38000 Grenoble, France; catherine.dejoie@esrf.fr
[4] Department of Chemistry, University of Zurich, 8057 Zurich, Switzerland; to dubravka.sisak@chem.uzh.ch
* Correspondence: martin.kuentz@fhnw.ch; Tel.: +41-61-228-5642

Received: 25 February 2019; Accepted: 5 April 2019; Published: 10 April 2019

Supporting Data

Powder X-ray diffraction patterns

The following graphs were used for the final formulation evaluation of the polyelectrolyte matrices. The following powder X-ray diffraction (PXRD) analyses were based on the benchtop X-ray diffractometer that is described in the methods section.

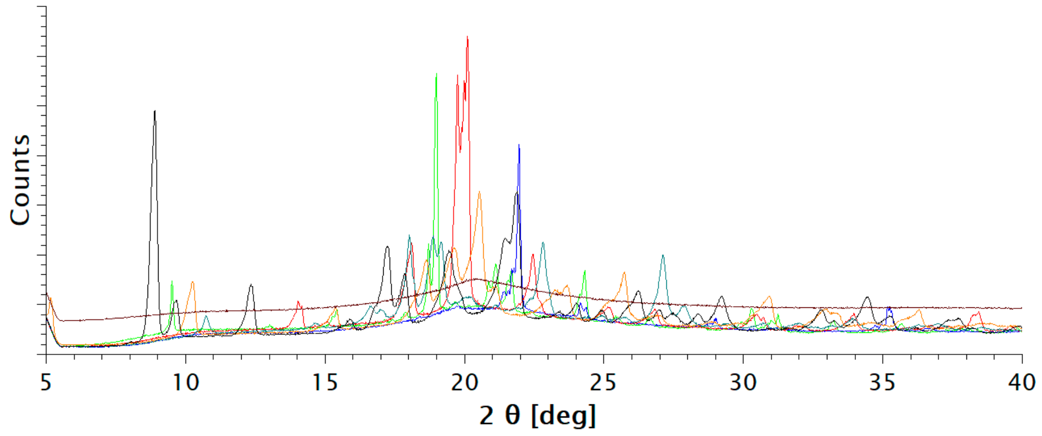

Figure S1. PXRD patterns of the physical mixtures. Colors and labels are in line with Figure 2 and 8, which can be inferred from the main article. The diffraction pattern of pure NaCMC in shown in bordeaux with an offset.

pharmaceutics

MDPI

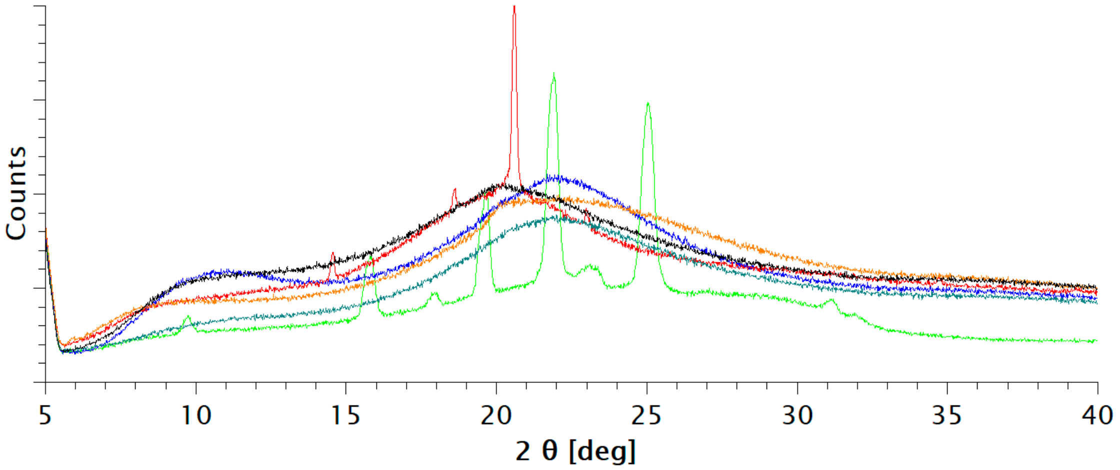

Figure S2. PXRD diffraction patterns of the solvent evaporates. Colors and labels are in line with Figure 2 and 8, which can be inferred from the main article.

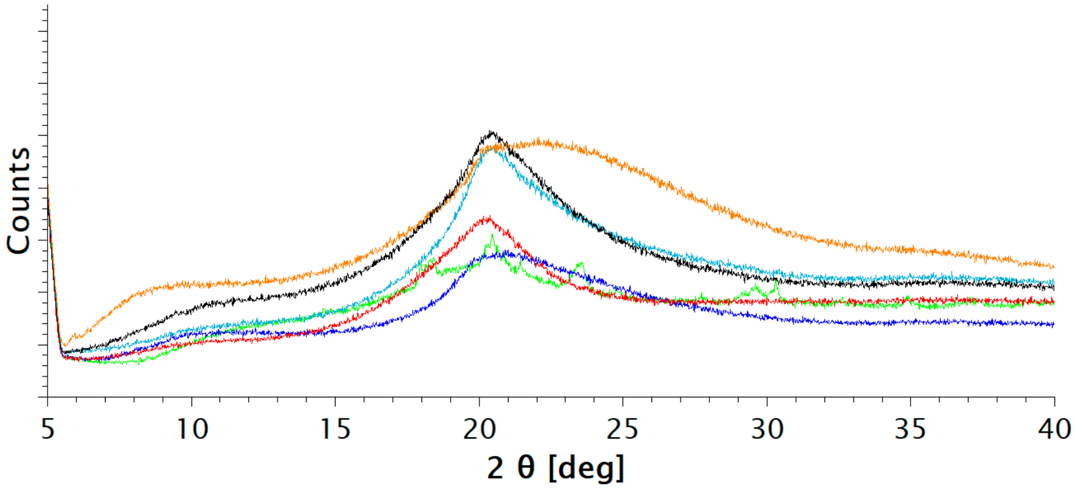

Figure S3. PXRD patterns of the extrudates. Colors and labels are in line with Figure 2 and 8, which can be inferred from the main article.

pharmaceutics

Hot stage microscopy and hot stage FTIR

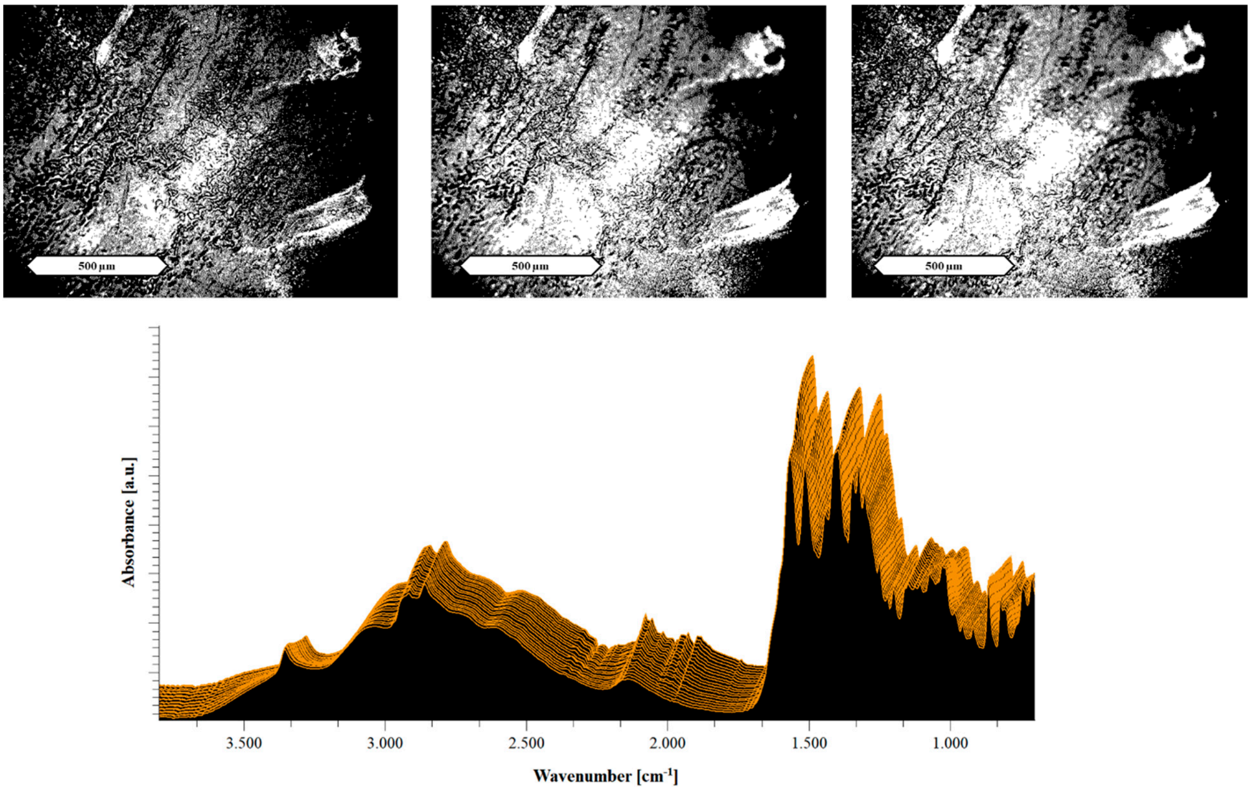

Figure S4. Heat resolved FTIR and HSM images of 50 % lysine / NaCMC solvent evaporates. The images at the top show HSM images taken at RT, 90 °C and 130 °C (from left to right). Each spectrum was measured at temperatures from 30°C (measured first in the front) to 130°C (in the back) by increasing steps of 5°C.

pharmaceutics

MDPI

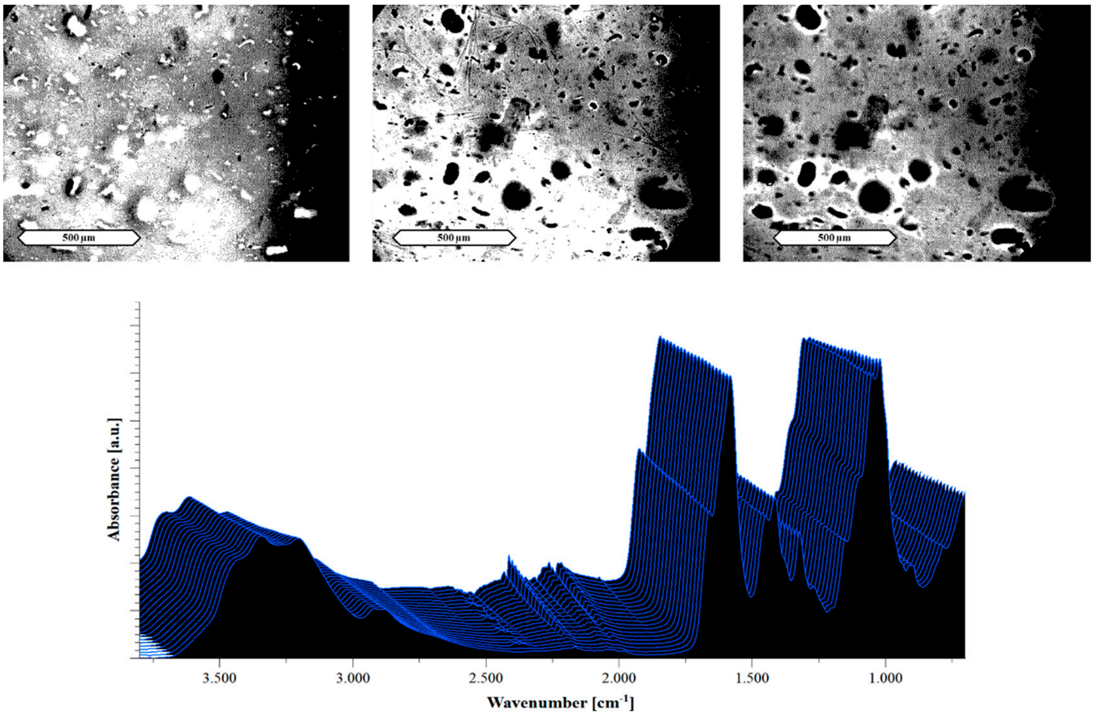

Figure S5. The images at the top show HSM images of 20 % urea / NaCMC solvent evaporated films taken at RT, 90 °C and 130 °C (from left to right). At the bottom, a temperature resolved FTIR spectrum is shown. Each spectrum was measured at temperatures from 30°C (measured first in the front) to 130°C (in the back) by increasing steps of 5°C
